# An Intelligent Cooperative Visual Sensor Network for Urban Mobility

**DOI:** 10.3390/s17112588

**Published:** 2017-11-10

**Authors:** Giuseppe Riccardo Leone, Davide Moroni, Gabriele Pieri, Matteo Petracca, Ovidio Salvetti, Andrea Azzarà, Francesco Marino

**Affiliations:** 1 Institute of Information Science and Technologies, National Research Council of Italy, 56124 Pisa, Italy; g.leone@isti.cnr.it (G.R.L.); gabriele.pieri@isti.cnr.it (G.P.); matteo.petracca@isti.cnr.it (M.P.); ovidio.salvetti@isti.cnr.it (O.S.); 2 Scuola Superiore Sant’Anna of Pisa, 56124 Pisa, Italy; andrea.azza@gmail.com (A.A.); fr.marino@santannapisa.it (F.M.)

**Keywords:** visual sensor networks, real time image processing, embedded vision, IoT middleware, internet of things, intelligent transportation systems, smart cities

## Abstract

Smart cities are demanding solutions for improved traffic efficiency, in order to guarantee optimal access to mobility resources available in urban areas. Intelligent video analytics deployed directly on board embedded sensors offers great opportunities to gather highly informative data about traffic and transport, allowing reconstruction of a real-time neat picture of urban mobility patterns. In this paper, we present a visual sensor network in which each node embeds computer vision logics for analyzing in real time urban traffic. The nodes in the network share their perceptions and build a global and comprehensive interpretation of the analyzed scenes in a cooperative and adaptive fashion. This is possible thanks to an especially designed Internet of Things (IoT) compliant middleware which encompasses in-network event composition as well as full support of Machine-2-Machine (M2M) communication mechanism. The potential of the proposed cooperative visual sensor network is shown with two sample applications in urban mobility connected to the estimation of vehicular flows and parking management. Besides providing detailed results of each key component of the proposed solution, the validity of the approach is demonstrated by extensive field tests that proved the suitability of the system in providing a scalable, adaptable and extensible data collection layer for managing and understanding mobility in smart cities.

## 1. Introduction

By 2050 over 70% of the world’s population will live in cities, metropolitan areas and surrounding zones. There is thus a strong interest in making our cities smarter by tackling the challenges connected to urbanization and high density population by leveraging modern Information and Communications Technologies (ICT) solutions. Indeed, progresses in communication, embedded systems and big data analysis make it possible to conceive frameworks for sustainable and efficient use of resources such as space, energy and mobility which are necessarily limited in crowded urban environments. In particular, Intelligent Transportation Systems (ITS) are envisaged to have a great role in the smart cities of tomorrow. ITS can help in making the most profitable use of existing road networks (which are not always further expandable to a large extent) as well as public and private transport by optimizing scheduling and fostering multi-modal travelling. One enlightening example of the role of ITS is represented by the seemingly trivial parking problem: it has been shown that a share of the total traffic of 10% (with peaks up to 73% [1]) is represented by cars cruising for parking spaces; such cars constrain urban mobility not only in the nearby vicinity of usual parking zones, but also in geographically distant areas, which are affected by congestion back-propagation. Indeed, the cruising-for-park issue is a complex problem which cannot be solely ascribed to parking shortage, but is also connected to fee policies for off-street and curb parking and to the mobility patterns through the city in different periods and days of the week. Indeed, cheap curb parking fees usually foster cruising, with deep impacts on global circulation, including longer time for parking, longer distances and a waste of fuel, resulting thus in incremented emissions of greenhouse gas. As such, the problem cannot be statically modeled since it has clearly a spatio-temporal component [2], whose description can be classically obtained only through detailed data acquisition assisted by manual observers which—being expensive—cannot be routinely performed. Nowadays, however, ITS solutions in combination with the pervasive sensing capabilities provided by Wireless Sensor Networks (WSN) can help in tackling the cruising-for-parking problem: indeed by the use of WSN it is possible to build a neat spatio-temporal description of urban mobility that can be used for guiding drivers to free spaces and for proposing adaptive policies for parking access and pricing [3].

More generally, pervasive sensing and ubiquitous computing can be used to create a large-scale, platform-independent infrastructure providing real-time pertinent traffic information that can be transformed into usable knowledge for a more efficient city thanks to advanced data management and analytics [4]. A key aspect for the success of these modern platforms is the access to high quality, high informative and reliable sensing technologies that—at the same time—should be sustainable for massive adoption in smart cities. Among possible technologies, probably imaging and intelligent video analytics have a great potential which has not yet been fully unveiled and that is expected to grow [5]. Indeed, imaging sensors can capture detailed and disparate aspects of the city and, notably, traffic related information. Thanks to the adaptability of computer vision algorithms, the image data acquired by imaging sensors can be transformed into information-rich descriptions of objects and events taking place in the city. In the past years, the large scale use of imaging technologies was prevented by inherent scalability issues. Indeed, video streams had to be transmitted to servers and therein processed to extract relevant information in an automatic way. Nevertheless, nowadays, from an Internet of Things (IoT) perspective it is possible to conceive embedded vision nodes having on-board suitable logics for video processing and understanding. Such recent ideas have been exploited in cooperative visual sensor networks, an active research field that extends the well-known sensor network domain taking into account sensor nodes enabled with vision capabilities. However, cooperation can be meant at different levels. For instance in [6] road intersection monitoring is tackled using different sensors, positioned in such a way that any event of interest can always be observed by at least one sensor. Cooperation is then obtained by fusing the different interpretation of the sensors to build a sort of bird’s eye view of the intersection. Instead in [7] cooperation among nodes is obtained by offloading computational tasks connected to image feature computation from one node to another. With respect to these previous works, one of the main contributions of this paper is the definition and validation of a self-powered cooperative visual sensor network designed for acting as a pervasive roadside wireless monitoring network to be installed in the urban scenario to support the creation of effective Smart Cities. Such an ad hoc sensor network was born in the framework of the Intelligent Cooperative Sensing for Improved traffic efficiency (ICSI) project [8], which aimed at providing a platform for the deployment of cooperative systems, based on vehicular network and cooperative visual sensor network communication technologies, with the goal of enabling a safer and more efficient mobility in both urban and highway scenarios, fully in line with ETSI Collaborative ITS (C-ITS). In this direction, and following the ICSI vision, the proposed cooperative visual sensor network is organized as an IoT-compliant wireless network in which images can be captured by embedded cameras to extract high-level information from the scene. The cooperative visual sensor network is responsible for collecting and aggregating ITS-related events to be used to feed higher levels of the system in charge of providing advanced services to the users. More in detail, the network is composed of new custom low-cost visual sensors nodes collecting and extracting information on: (i) parking slots availability, and (ii) traffic flows. All such data can be used to provide real-time information and suggestions to drivers, optimizing their journeys through the city. Further, the first set of collected data regarding parking can be used in the Smart City domain to create advanced parking management systems, as well as to better tune the pricing policies of each parking space. The second set of data related to vehicular flows can be used for a per-hour basis analysis of the city congestion level, thus helping the design of innovative and adaptive traffic reduction strategies.

Extraction and collection of such ITS-related data is achieved thanks to the introduction of novel lightweight computer vision algorithms for flow monitoring and parking lot occupancy analysis, which represent another important contribution of this paper; indeed, the proposed methods are compared to reference algorithms available in the state of the art and are shown to have comparable performance, yet they can be executed on autonomous embedded sensors. As a further point with respect to previous works, in our proposal, cooperation is obtained by leveraging a Machine-2-Machine (M2M) middleware for resource constrained visual sensor nodes. In our contribution, the middleware has been extended to compute aggregated visual sensor node events and to publish them using M2M transactions. In this way, the belief of each single node is aggregated into a network belief which is less sensitive either to partial occlusion or to the failure of some nodes. Even more importantly, the visual sensor network (and the gain in accuracy that is possible to obtain thanks to the cooperative approach) is not only proved through simulation or limited experimentation in the lab, but is shown in a real, full-size scenario. Indeed, extensive field tests showed that the proposed solution can be actually deployed in practice, allowing for an effective, minimally invasive, fast and easy-to-configure installation, whose maintenance is sustainable, being the network nodes autonomous and self-powered thanks to integrated energy harvesting modules. In addition, during the tests, the visual sensor network was capable of gathering significant and precise data which can be exploited for supporting and implementing real-time adaptive policies as well as for reshaping city mobility plans.

The paper is organized as follows. Related works are reviewed in Section 2, focusing both on architectural aspects and computer vision algorithms for the targeted ITS applications. In Section 3 the main components used for building the cooperative visual sensor network are introduced and detailed, while in Section 4, the findings of the experiments for the validation of the embedded computer vision logics (Section 4.1) and of the IoT-compliant middleware for event composition (Section 4.2) are reported together with the global results of field tests (Section 4.3). Section 5 ends the paper with ideas for future research.

## 2. Related Works

With the increasing number of IoTdevices and technologies, monitoring architectures have moved during the years from *cloud* based approaches towards *edge* solutions, and more recently to *fog* approaches. The main drivers of this progressive architectural change have been the capabilities and complexities of IoT architectural elements. As the computational capacity of devices has increased, the intelligence of the system has been moved from its core (cloud-based data processing) to the border (edge-computing data analysis and aggregation), pushing further until reaching devices (fog-computing approach) to spread the whole system intelligence among all architectural elements [9]. One of the main features of fog computing is the location awareness. Data can be processed very close to their source, thus letting a better cooperation among nodes to enhance information reliability and understanding. Visual sensor networks in the ITS domain have been envisioned during the years as an interesting solution to extract high value data. The first presented solutions were based on an edge-computing approach in which whole images or high-level extracted features were processed by high computational nodes located at the edge of the monitoring system. Works such as [10,11] are just an example of systems following this approach. More recent approaches leverage powerful visual sensor nodes, thus proposing solutions in which images are fully processed on-board, thus exploiting fog-computing capabilities. Following this approach, several works have been proposed in the literature by pursuing a more implementation-oriented and experimental path, works such as [12,13] must be cited in this line of research, and, more recently a theoretical and modeling analysis [14]. By following a fog computing approach, the solution described in this paper proposes (i) a visual sensor network in which the logic of the system is spread among the nodes (visual sensors with image processing capabilities), and where (ii) information reliability and understanding is enhanced by nodes cooperations (cooperative middleware) exploiting location awareness properties.

As discussed in the Introduction, the proposed visual sensor network is applied to two relevant ITS problems in urban mobility, namely smart parking and traffic flow monitoring. Nowadays, besides counter-based sensors used in off-street parking, most smart parking solutions leverage two sensor categories, i.e., in situ *sensors* and *camera-based sensors*. The first category uses either proximity sensors based on ultrasound or inductive loop to identify the presence of a vehicle in a bay [15,16]. Although the performance and the reliability of the data provided by this kind of sensors are satisfactory, nevertheless installation and maintenance costs of the infrastructure have prevented massive uptake of the technology, which has been mainly used for parking guidance systems in off-street scenarios. Camera-based sensors are based on the processing of videos streams captured by imaging sensors thanks to the use of computer vision methods. In [17] two possible strategies to tackle the problem are identified, namely the *car-driven* and the *space-driven* approaches. In car-driven approaches, object detection methods, such as [18], are employed to detect cars in the observed images, while in space-driven approaches the aim is to asses the occupancy status of a set of predefined parking spaces imaged by the sensor. Change detection is often based on background subtraction [19]. For outdoor applications, background cannot be static but it should be modeled dynamically, to cope with issues such as illumination changes, shadows and weather conditions. To this end, methods based on Gaussian Mixture Models (GMM) [20] or codebooks [21] have been reported. Other approaches are based on machine learning, in which feature extraction is followed by a classifier for assessing occupancy status. For instance, in [22], Gabor filters are used for extracting features; then, a training dataset containing images with different light conditions is used to achieve a more robust classification. More recently, approaches based on textural descriptors such as Local Binary Patterns (LPB) [23] and Local Phase Quantization (LPQ) [24] have appeared. In [25], which can be considered as the state of the art, Support Vector Machines (SVM) [26] are used to classify parking space status on the basis of LPB and LPQ features and an extensive performance analysis is reported. Deep learning methods have also been recently applied to the parking monitoring problem [27]. All the previously described methods are based on the installation of a fixed camera infrastructure. Following the trends in connected and intelligent vehicles, however, it is possible to envisage novel solutions to the parking lot monitoring problem. For instance, in [28], an approach based on edge computing is proposed in which each vehicle is endowed with a camera sensor capable of detecting cars in its field of view by using a cascade of classifiers. Detections are then corrected for perspective skew and, finally, parked cars are identified locally by each vehicle. Single perceptions are then shared through the network in order to build a precise global map of free and busy parking spaces. A current drawback is that the method can provide significant results and a satisfactory coverage of the city only if it is adopted by a sufficient number of vehicles. Similarly, several methods based on crowd-sourcing [29] have been reported in the literature, most of which rely on location services provided by smart-phones for detecting arrivals and departures of drivers by leveraging activity recognition algorithms [30,31].

For what regards traffic flow monitoring, the problem has received great attention from the computer vision community [32], even for the specific case of urban scenario [33]. Nevertheless most of the existing methods use classical computer vision pipelines that are based on background subtraction followed either by tracking of the identified blobs or of the detected vehicles (see, e.g., [34]). Such approaches are too demanding in terms of computational resources for deployment on embedded sensors.

Among the various approaches and algorithms used, only few of them are for a real-time on site processing: among them, Messelodi et al. in [35] perform a robust background updating for detecting and tracking moving objects on a road plane, identifying different classes of vehicles. Their hardware is not reported, but in any case the heavy tracking algorithm let understand that it cannot be an embedded and autonomous platform. A similar approach is reported in [36] where the tracking is based on Optical-Flow-Field estimation following an automatic initialization (i.e., localization), but the final goal is more related to a 3-D tracking and tests were performed on a laboratory PC. Another feature-based algorithm for detection of vehicles at intersection is presented in [37], but again the used hardware is not reported, and the tests seem to be performed only on lab machines. An interesting embedded real-time system is shown in [38], for detecting and tracking moving vehicles in nighttime traffic scenes. In this case, they use a DSP-based embedded platform operating at 600 MHz with 32 MB of DRAM, their results on nighttime detection are very good, but yet their approach did not have to cope with low-energy constraints. In [39], the real-time classification of multiple vehicles, also performing a tracking based on Kalman filtering, is performed using commercially available PCs, yet results are around 95%. Lai et al. [40] propose a robust background subtraction model facing lighting changes problems with slowly moving objects in an *acceptable* processing time. However, the used hardware is not described and the final acceptable processing frame rate is around 2.5 fps, which is not acceptable for a normal traffic condition. The same problem arises in [41], where they perform real-time vision with so-called *autonomous tracking units*, which are defined as powerful processing units. Their test-bed are parking (in particular airport parkings) with slow moving vehicles and their final processing rate is again very low i.e., below 5 fps. Finally, in [42] the algorithm based on Support Vector is not used in real-time condition and, even if the processing times and the hardware are not reported, it must be a powerful unit, yet obtaining around 95% accuracy.

## 3. System Architecture and Components

In this section, the system architecture is reported before presenting the prototyped visual sensor node and the two key components that enable the creation of a cooperative visual sensor network: namely, computer vision logics, especially designed for deployment on embedded sensors, and the IoT middleware. The vision logics are meant to be deployed on board each single sensor in the visual sensor network, which is then able to provide autonomously its interpretation of the scene at *sensor level*. These local processing results are then integrated and converted into a more advanced, robust and fault tolerant understanding of the scene at *network level* [43], leveraging a middleware layer capable of event composition over resource constrained sensor networks. Both the computer vision logics and the middleware solution have been used in the design of a visual sensor network, that has been physically deployed and validated on the smart camera prototype described in Section 3.1.

### 3.1. System Architecture and Visual Sensor Prototype

**Figure 1:**
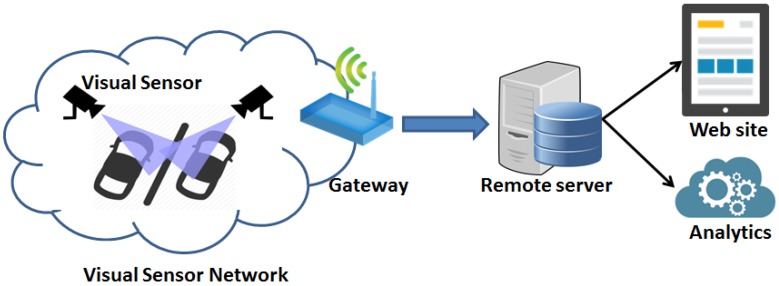
System architecture.

The high level system architecture of the deployed monitoring sensor network for urban mobility is reported in Figure 1. It is mainly composed of three components: (i) the visual sensor devices able to exploit on board processing capabilities of the scene while providing M2M communication, (ii) the system gateway, acting as connection point between devices belonging to the visual sensor network and the Internet world, and (iii) the remote server in which detected events and data are stored for both analytic purposes and visualization on web portals. The following of the section focuses on the monitoring part of the system by reporting motivation and design choices behind the realization of the visual sensor node.

Although many nodes that might support the creation of a visual sensor network are currently available (see, e.g., [44,45] for a review of some of them), nevertheless none seems to be satisfying for targeting outdoor ITS applications and to have capabilities for M2M communication. Actually, M2M communication is seen to be a key aspect to drive the shift from classical cameras and centralized video processing architecture to smart cameras and distributed video analytics over heterogeneous sensor networks. For these reasons, the design of an integrated IoT node was addressed, taking into account several requirements both from the functional and non-functional perspective. Indeed, the node should have enough computational power to accomplish the computer vision task envisaged for urban scenarios as described in Section 3.2 but, at the same time, it should be based on low power and low cost technologies. In this way, the nodes might be used to setup an autonomous, self-powered network in the city, using whenever possible photo-voltaic panels or other energy harvesting opportunities. Low cost components and architecture, in addition, guarantee that –once engineered– the node can be manufactured at low cost in large quantities, which is a fundamental aspect for the sustainability and wide scale adoption of the proposed technology. As for network communication, the node should be ready to support the interfaces needed in the IoT domain and, in particular, to support the middleware described in Section 3.3.

Inside the node, two main logical components have been identified, corresponding to networking and vision aspects. In particular the *networking component* takes care of communication both by managing M2M transactions and by interacting with the vision processes, e.g., by requesting their activation, transferring of their results, or setting parameters and behaviors of computer vision algorithms. Thus, networking component needs to be operating most of the time to guarantee responsiveness of the node to incoming requests and it must be low-consuming, but no high computational resources are needed. By converse, the vision component consumes many computational and energetic resources to run the algorithms reported in Section 3.2 when the node is operational; however, if there is resource shortage, policies might be adopted to slow down computation, entering eventually a best-effort behaviour without affecting the overall functioning of the sensors.

It is worthwhile to notice that the visual sensor network is intrinsically privacy-preserving. Indeed, images are processed locally at sensor level, without the need to transfer them to other locations, and then disregarded. Therefore, confidential information contained in the acquired images is not at risk, since they are neither stored nor accessible from a centralized location. Although it was not a primary scope of this paper (and, thus, it has not been taken into account in the implementation), a further security level on the communications inside the visual sensor network can be added; for instance, security concerns might be addressed using the methods proposed in [46], where an elliptic curve cryptography approach for IoT devices is adopted and applied to the smart parking domain.

For the realization of the new node, a custom printed circuit board (PCB) has been designed to have the maximum flexibility of use while maximizing the performance/consumption ratio. A good compromise has been achieved by using a *Freescale CPU* based on the ARM architecture, with support for MMU-like operating systems GNU/Linux. This architecture has the advantage to integrate within it a PMU (Power Management Unit), in addition to numerous peripheral interfaces, thus minimizing the complexity of the board. Moreover, the CPU package of type TQFP128 allowed to minimize the layout complexity, since it was not necessary to use multilayer PCB technologies for routing. Thus, the board can be printed also in a small number of instances. The choice has contributed to the further benefit of reducing development costs, in fact, the CPU only needs an external SDRAM, a 24 MHz quartz oscillator and an inductance for the PMU. Also considering the footprint of running programs, a 64 MB SDRAM has been selected, which gives the possibility to keep in main memory a number of full resolution images more than sufficient for addressing the targeted applications. The chosen architecture has been proved to have an average consumption measured at the highest speed (454 MHz) less than 500 mW, which makes it suitable for using energy harvesting strategies. A microSD slot is present, which is essential for booting the system, booting the kernel and file-system associated (EXT4); the board can be upgraded simply by changing the contents of the microSD. The PCB is connected to a SEED-EYE device [47] for managing networking aspects and has the capability to integrate camera sensors supporting USB Video Class device (UVC). The selection of a low-cost device brought to an easy-to-buy and cheap camera, the *HP HD 2300 Webcam*, that has been used during the experimentation.

### 3.2. Embedded Vision Logics for Visual Sensor Networks

In the proposed cooperative visual sensor network, each node consists of an embedded sensor equipped with vision logics able to perform real-time scene understanding. The goal of this analysis is two-fold: (i) exact detection of parking slot availabilities in a parking lot, and (ii) traffic flow analysis on relevant roads for parking. The major issue described here is the balance between the need to take into account low cost, scalability, and portability requirements, and the production of reliable and efficient computer vision technologies deployable on an IoT smart object.

Among the various scenarios, the differences in specific requirements are substantial: real-time constraints for traffic flow versus so-called near real-time (i.e., processing fulfilled in temporal terms of minutes) for parking slot monitoring; smaller area of interest to monitor a two-lane road, wider area to monitor a parking lot with several spaces; need for a fast frame acquisition rate for performing an efficient traffic flow monitoring. In the following, the two specific scenarios and the solutions implemented for solving them are analysed separately.

#### 3.2.1. Parking Lot Availability Scenario

**Figure 2:**
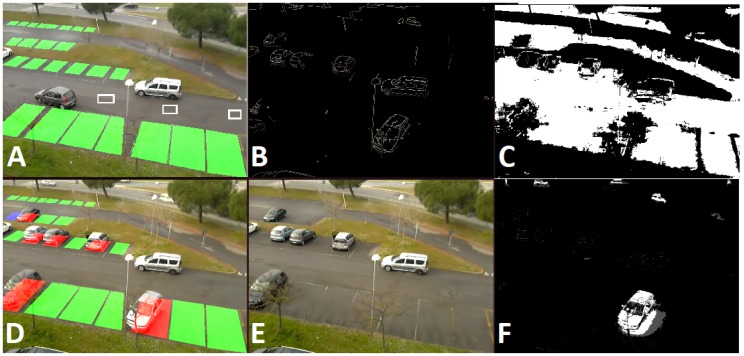
(**A**) RoI for a set of parking lots are set up manually with the help of a graphic tool. Small rectangles on the driveway define the samples for asphalt detection (**B**) The output of the Canny edge detector (**C**) White regions represent areas where asphalt is detected (**D**) Current input image with augmented reality displaying the status (**E**) Background image (**F**) Frame differencing to detect major status changes.

As discussed in Section 2, the car-driven and the space-driven approaches are the two main strategies to deal with the problem of detecting parking lot vacancies. In car-driven approaches, features detection methods are employed to detect cars in the observed images, while in space-driven approaches the aim is to detect empty spaces rather than vehicles. Recent works proposed in the literature show very good performance (see Table 2 in the next section). Although this scenario, as mentioned above, allows for less restrictive processing constraints (e.g., not a strict real-time processing), state-of-the-art algorithms generally require performing hardware and it is not possible to deploy them on low memory/low computational power sensors. Various algorithms have been studied and designed to be deployed on the proposed visual sensor network for the analysis of parking lot occupancy status. The methodology chosen is based alternatively on the analysis of single frames, or on frame differencing, in order to highlight the changes in the Regions of Interest (RoI) with respect to an adaptive GMM background reference image [48]. For outdoor applications, background cannot be static but it should be modeled dynamically, to cope with issues such as illumination changes, shadows and weather conditions. In order to improve the robustness of the algorithm with respect to environmental light changes, normalized versions of the images are computed and used, with respect to global illumination parameters (average and variance of both the current and reference image). To improve computational efficiency, image analysis and frame differencing for detecting changes are performed only on predetermined RoI in the acquired frame. Each of the RoI corresponds to a specific parking slot, it is set up manually with the help of a graphic tool, and can be of any polygonal shape (this helps to avoid occlusion like trees, poles or other static artifacts). In Figure 2A) the green zones are the defined parking lots RoI. For each of the regions a confidence value of the occupancy is computed in real-time. The output of a single node is a vector of probability values of the occupancy for each parking slot.

In the first phase, the input image is evaluated with respect two lightweight algorithms (a car-driven approach and a space-driven one). The car-driven method searches for car features in the predefined RoI. A fast edge-detector, the Canny operator [49] is used to obtain a very crisp image of the vehicles contours of the frame acquired at time *t* as it is shown in Figure 2B; we guess if a vehicle occupies the RoI $R_{k}$ calculating the index $e_{k}\left(t\right)$ which is proportional to the ratio of edge pixels with respect to the square root of total number of pixels in $R_{k}$, i.e.,:

(1) $$e_{k}\left(t\right)=\#\left(\mathrm{edge}\;\mathrm{pixels}\;\mathrm{in}\;R_{k}\right)\;/{\#(\mathrm{pixels}\;\mathrm{in}\;R_{k})}^{\left(1/2\right)}$$

For an empty slot $e_{k}\left(t\right)$ is very close to zero, while slots with active edges (i.e., most probably not empty) have a higher value. This index cannot be used if a pole or a tree partially create an occlusion of the slot (they can be misinterpreted as a presence in the lot).

The second index comes out from a space-driven approach: considering that an empty slot should appear as plain asphalt concrete, we aim to detect asphalt areas based on the color characteristics of small samples on the driveway (see Figure 2A); we consider a subset of Hue and Saturation in the HSV color space (we discard the brightness to be more robust against different illumination); furthermore these values are periodically updated to include changes occurring due to time/light changes, rain, etc. For every input frame, the asphalt detection acts like a binary filter (see Figure 2C)

The index $a_{k}\left(t\right)$ is the ratio of asphalt pixels and total pixels of a RoI, i.e.:

(2) $$a_{k}\left(t\right)=\#\left(\mathrm{asphalt}\;\mathrm{pixels}\;\mathrm{in}\;R_{k}\right)\;/\#\left(\mathrm{pixels}\;\mathrm{in}\;R_{k}\right)$$

This index is really trustworthy if it is close to 1. As a rule of thumb for value greater than 90% the lot can be consider available. The index of a not-empty lot is generally below 20% but cars with similar color of the asphalt sometimes arrive at higher value. However the presence of different non gray area like windows, tires, plates and so on do never scores like plain asphalt.

Combining these two indexes, we compute a final evaluation of occupancy probability $P_{k}\left(t\right)$ of the slot *k* as seen from this sensor:

(3) $$P_{k}\left(t\right)=e_{k}\left(t\right)*\left(1-a_{k}\left(t\right)\right)$$

This joint index is valuable mainly to determine the initial status (analysis of the initial frames) or when there is a sudden light change that blinds the camera. After the first *k* frames (being *k* a number between 10 and 30) the background image is available (see Figure 2E) and from that moment all the major events are determined by frame differencing. This is a very reliable detector also with our 0.5 fps because during parking every vehicle move is slow (Figure 2F shows the very clear shape of the parking car—notice the absence of this car in the background image). If a major change happens in a RoI, it is very easy to be detected and the system keeps track of the history of these changes.

#### 3.2.2. Traffic Flow Monitoring Scenario

On the contrary to the previous scenario, in this one restrictive processing constraints exist, due to the need to detect not only all the passing vehicles but also in view of a deeper analysis of the traffic flow, i.e., sensing average speeds and categories of vehicles.

With respect to the classical computer visions techniques reviewed in Section 2, an ad hoc lightweight method was designed which is suitable for deployment on embedded devices.

For the deployment on the visual sensor network, background detection is performed only on small quadrangular RoI which are sufficient for modelling physical rectangles under a perspective skew. In addition, lightweight methods are implemented for background modelling, that is determining if a pixel belongs to foreground (i.e., meaning that it is changed w.r.t. the previous scene), or to the background (i.e., pixel unchanged). Three main approaches have been considered, i.e., frame differencing, static background and adaptive background. The latter class of algorithms proved to be the most robust for use in uncontrolled outdoor scenes. The background is constantly updated using both the previous background model and the latest acquired actual image.

The data extraction procedure starts by taking as input one of several RoI for each lane suitably segmented in foreground/background. When processing the frame acquired at time *t*, the algorithm decides if the RoI $R_{k}$ is occupied by a vehicle or not. Such an occupancy test is based on the ratio of pixels changed with respect the total number of pixels in $R_{k}$, i.e.,:

(4) $$a_{k}\left(t\right)=\frac{\#(\mathrm{changed}\;\mathrm{pixels}\;\mathrm{in}\;R_{k})}{\#(\mathrm{pixels}\;\mathrm{in}\;R_{k})}$$

**Figure 3:**
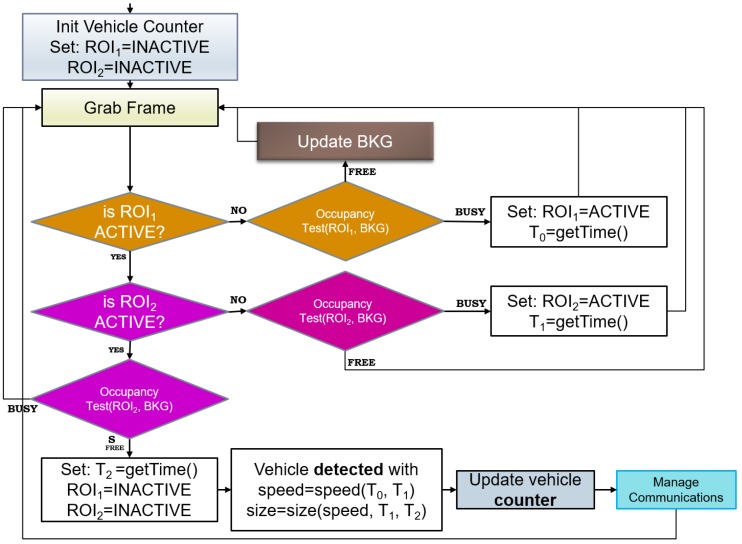
Flow chart of the traffic flow monitoring algorithm.

Then $a_{k}\left(t\right)$ is compared to a threshold τ in order to evaluate if a vehicle was effectively passing on $R_{k}$. If $a_{k}\left(t\right)>\tau$ and at time t−1 no vehicle was detected, then a new transit event is generated. If a vehicle was already detected instead at time t−1, no new event is generated but the time length of the last created event is incremented by one frame. Finally, when at a time t+j no vehicle is detected (i.e., $a_{k}\left(t+j\right)<\tau$), the transit event is declared as accomplished and no further updated. Assuming that the vehicle speed is uniform during the detection time, the number of frames *j* in which the vehicle was observed is proportional to the vehicle length and inversely proportional to its speed. In the same way, it is possible to use two RoI, $RoI_{1}$ and $RoI_{2}$, lying on the same lane but translated by a distance Δ, to estimate the vehicle speed. The algorithm uses two RoI for classifying vehicles with respect to their size and speed class (see Figure 3). At the beginning both RoI are set as inactive. Then a frame is grabbed from the sensor and an occupancy test is run on both RoI sequentially. If a RoI becomes busy according to the occupancy test, it is marked as active. $RoI_{2}$ is only tested for occupancy when $RoI_{1}$ is active. When both RoI are active, a transit event has occurred. The algorithms continues to grab frames until $RoI_{2}$ becomes free. At this point, the transit event is concluded since the vehicle has left the detection area. It is then possible to classify the event. Using counters based on the elapsed time and knowing the distance Δ among the RoI, the vehicle speed and size are computed. Notice that the main loop described is a simplified version of the one actually implemented, where there are some further controls and heuristics to avoid false alarms. For instance, a timeout is set for the $RoI_{2}$ to become active after $RoI_{1}$ has. Indeed, since the RoI are within a few meters distance, no more than some seconds can elapse from the occupation of the first to the occupation of the second one.

### 3.3. IoT Middleware for Event Composition

In an ITS system, in which the roadside network is composed of an IoT-compliant visual sensor network devices, the remote management of the nodes as well as their cooperation and data collection functionalities can be all managed by an IoT middleware. In this respect the ICSI Middleware proposed in [50] has been extended and adapted to forward both simple and aggregated visual sensor node events towards remote gateways by using Machine-2-Machine (M2M) transactions [51]. Further, the middleware enables a remote configuration of the IoT visual sensor nodes, which turns out to be useful in several scenarios, for example when event aggregation strategies are adopted. The possibility of aggregating events in the roadside segment enables in-network processing capabilities which can be used to increase the robustness of the information. In fact, by using visual sensor nodes a possible problem is the temporary occlusion of the field of view, which can be overtaken deploying different visual sensors monitoring the same scene, while requiring an event processing heuristic inside the network. When an event, e.g., a parking slot becoming free or busy, is detected by more than a single sensor it is necessary to process and aggregate all the events to provide a final decision on the status of the slot. In this depicted scenario the node responsible for the in-network event aggregation could be a single point of failure in case it fails, e.g., due to battery depletion. To tackle this problem the middleware implements a dynamically reconfigurable system, based on standard interfaces, capable of moving event aggregation tasks from node to node at runtime. Such a functionality, better described in the following, is implemented by enabling a virtual machine based approach in the part of the visual sensor node responsible for the IoT communications.

**Figure 4:**
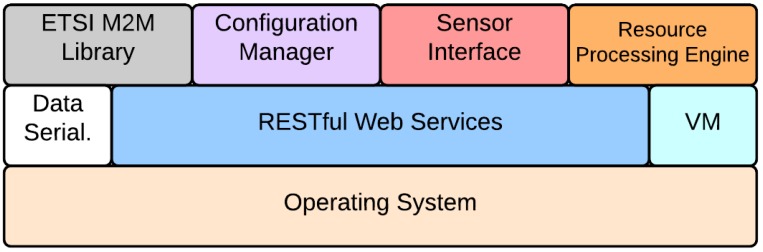
Middleware architecture.

Considering a pervasive roadside segment based on visual sensor nodes, the ICSI Middleware is a software component instantiated on each node. The high level middleware architecture is shown in Figure 4. The middleware has been designed following the Component-based Software Engineering approach, which promotes the Separation of Concerns and the Single Responsibility principles. According to these principles, the ICSI middleware has be partitioned into distinct modules which encapsulate very specific, not overlapping functionalities, and which communicate with each other via interfaces. The adoption of this approach allowed to come up with a high cohesive and low coupled system, with all the implied benefits in terms of maintainability and flexibility. The core modules are placed on-top of the operating system and make use of networking, virtualization and data serialization services provided by embedded software libraries. The main middleware components are: (i) the Resource Processing Engine (RPE), taking care of in-network event processing tasks; (ii) the ETSI M2M library, supporting standard communication with system gateways; (iii) the Sensor Interface, a software library abstracting the functionality of on-board sensors; (iv) the Configuration Manager, enabling the remote configuration of the node.

The whole middleware has been developed on top of Contiki OS [52], an open-source operating system targeted to IoT compliant devices and fully supporting IoT protocols, i.e., 6LoWPAN [53,54], RPL [55], and CoAP [56]. Contiki has been preferred to other embedded operating systems such as Riot and TinyOS because of its full and certified implementation of the IoT stack. In order to support some of the advanced features of the ICSI RPE, we added client-side support for CoAP Observe, which is needed by RPE to “observe” the input resources. Moreover we implemented IPv6 loopback communication (i.e., the possibility for a process to communicate with another process on the same node using sockets), needed by the RPE tasks to interact with resources belonging to the node on which they are deployed. The RESTful Web Service [57] component, based on the CoAP protocol, handles all network data inputs, outputs and implements a resource directory service. Such a component uses other important features implemented in the CoAP implementation provided by Contiki: (i) block-wise transfers to manage large resource representations; (ii) resource observation, to publish changed resource representation to all subscribers; (iii) sending of the acknowledgment for a request and the relative application data in separate responses in order to avoid undesired request retransmissions and timeout when the request involves time consuming tasks; (iv) resource discovery using the CoRE Link Format by automatically generating the /well-known/core handler at which all resources are registered. The middleware virtualization capabilities have been integrated by using PyMite [58], a lightweight Python interpreter on which tasks and applications can be instantiated at run-time by uploading Python bytecodes. Python has been preferred to other commonly used interpreted programming languages such as Java and Javascript, because the former does not allow the definition of processing functions by means of scripts and the latter cannot be compiled into bytecode, and both these characteristics do not fit well in constrained scenarios. PyMite has been specially designed to be executed on devices with scarce resources, e.g., microcontrollers with limited Flash and RAM capabilities. The Data Serialization Library component provides data serialization services in the EXI format, a very compact representation of XML, which provides better performance in compressing M2M messages [59].

The in-network event composition is performed by the RPE module. Such a component is placed on top of the virtualization module, and it is based on T-Res [60], a framework enabling in-network processing in IoT networks.

**Figure 5:**
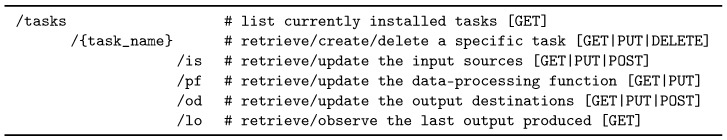
T-Res interface.

T-Res comes with the CoAP-based RESTful interface depicted in Figure 5, which allows to remotely instantiate, configure and delete tasks on a node. T-Res tasks are defined by their input sources (/is), the data processing they perform (/pf), and the destination of their output (/od). According to the RESTful paradigm the input sources and the output destination are CoAP resources specified by means of their Uniform Resource Identifiers (URIs), while the processing function resource /pf contains the Python bytecode of the task to be executed. It is possible to dynamically modify the behavior of a task by invoking the proper CoAP methods on such resources. Specifically, it is possible to change the processing function bytecode of a task simply performing a PUT on the relative /pf subresource providing the new bytecode. By allowing the run-time instantiation of tasks as Python bytecodes, the event processing function can be moved at run-time from one node to another, e.g., to a node having a better connectivity or a larger residual energy, completely decoupling the source of the information from the single physical sensor. The Configuration Manager is the component responsible for the global configuration of the ICSI Middleware. Each configurable component is registered through the RESTFUL Web Service and provides the APIs to allow remote configuration. This component provides access to a wide range of functions, including: (i) RPE configuration (input/output resources and processing); (ii) Over-the-air software update; (iii) Energy Policy Reconfiguration (duty cycle configuration); (iv) Networking and general purpose maintenance features. The ETSI M2M library uses the services provided by the data serialization libraries and exposes a set of APIs to encode/decode M2M messages according to ETSI specifications.

## 4. Results

The proposed visual sensor network was tested separately for the vision and networking parts in order to validate each single logical component; then the prototype was actually installed in the city of Pisa for an extensive field trial.

### 4.1. Evaluation of Embedded Vision Logics

In this section, tests and evaluations of the algorithms for traffic and parking monitoring are presented. These first tests were performed in order to be able to deploy the algorithm on the actual visual sensor network, through an assessment of the performance. Processing of these data was performed off-line in lab, but an important note is that all the data used were real-time acquired data from the real world.

#### 4.1.1. Parking Lot Availability Tests

**Figure 6:**
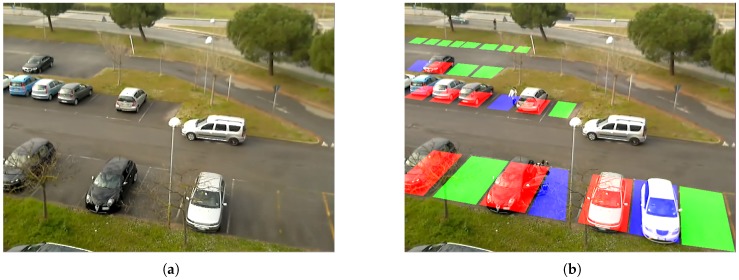
Example of parking lot analysis: background model at time *t* (**a**) and real-time output at time *t* (**b**).

Following the description of the background modelling given in Section 3.2.1, in Figure 6 the background model at a specific frame *t* of the acquired test sequence is shown (Figure 6a) and its corresponding processed output (Figure 6b). In the processed frame the red colour means *busy* because the belief of occupation is very strong due to the high number of edges and low asphalt detection inside the RoI. On the contrary, green parking slots indicate the status *available* because a very large area of asphalt is found within and few pixels come out from the edge detector. The blue colour expresses *uncertainty* because the combination of edge and asphalt beliefs is between the two thresholds of the hysteresis. In this latter situation, no change occurs in the status, i.e., the final output remains the same of the one given for the previous frame. There is a difference between the right-most blue slot and the others: in the bottom right slot, there is such an uncertainty because the vehicle has just arrived and it is not integrated in the background yet as can be seen from the picture on the left. The asphalt detection considers the background image (for stability reason), so only when the vehicle is part of it (i.e., after about 30 frames) the colour will change to red. The uncertainty condition of the other three slots are due to the partial occupation of the slot by entities that are not big enough to be classified as a vehicle (e.g., a person walking in and parts of vehicles from alongside slots).

In order to evaluate the performance of the method various video sequences were acquired during different times of the day (i.e., with different light conditions) and a comparison was made between the log of algorithm output versus a manually annotated ground truth, i.e., acquired frames were manually pointed out when a status change happened, that is an available parking lot became busy or the contrary. The Overall Error Rate (OER) is the metric proposed in [25] and is defined as the ratio of total errors (i.e., False Positive plus False Negative) and the total responses (i.e., False Positive plus False Negative plus True Positive plus True Negative):

(5) $$\mathrm{OER}=\frac{\mathrm{FP}+\mathrm{FN}}{\mathrm{FP}+\mathrm{FN}+\mathrm{TP}+\mathrm{TN}}$$

Notice that in an outdoor scenario (which is the setting of the experimentation carried out in the paper), even in absence of parking status changes, there are a number of non stationary elements that might interfere with the algorithm. These include for instance light changes, casted shadows and temporary occlusions. Furthermore in order to compare the system with others that operate at single frame level (their output is the state of each slot based on the analysis of a single image) it seems reasonable to consider every single frame output as the complete set of the slots’ status; thus the denominator of the ratio corresponds to the multiplication of the slots monitored and the total frames considered:

(6) $$\mathrm{ErrorRate}=\frac{\sum_{i=1}^{\mathrm{TotalFrames}}\left({\mathrm{FP}}_{i}+{\mathrm{FN}}_{i}\right)}{\mathrm{Total}\mathrm{Frames}\;\cdot \;\mathrm{Total}\mathrm{Slots}}$$

where ${\mathrm{FP}}_{i}+{\mathrm{FN}}_{i}$ is the total number of errors made when analyzing frame *i*.

In Table 1, the results of several sample acquired sequences from separate cameras are presented.

The average error rate based on five different cameras is 0.65%. Although a number of papers in literature has been presented on the topic, an accurate comparison is difficult to be proposed, since most of the approaches are far from being based on a “low-cost low-consumption embedded platform”, yet their performance are not radically different from the one achieved by our method. A comparison with some related work can be found in Table 2.

In particular, up to the best of our knowledge [25] is considered to be the most performant approach in the state of the art; it is based on complex features (LPB and LPL) and it is target for high end computer. Recently [27] showed good result using deep learning on a smart camera based on Raspberry Pi platform; nevertheless the training phase of the network has been executed offline with performing hardware and the necessary memory size to use the Convolutional Neural Network is ten times the one available on the visual sensor node proposed in this paper; furthermore the system proposed in [27] needs about 15 s to analyze an image and calculate its output, while our system achieves the result in 2 s.

#### 4.1.2. Traffic Flow Monitoring Tests

**Figure 7:**
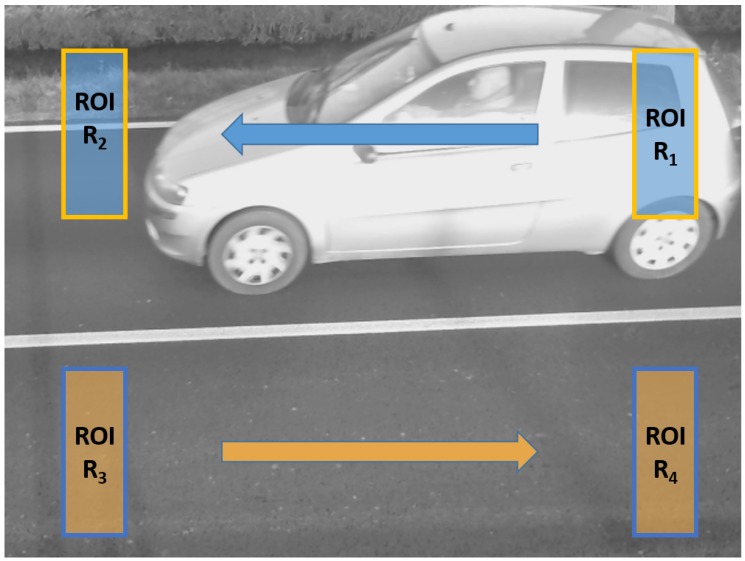
Traffic flow analysis: view from sensor test set-up and example of vehicles transit in the field of view of the sensor, which may cause occlusion to the upper lane.

Traffic monitoring data were acquired using a temporary installation of the sensors on the same site of the final deployment, so that test sequences acquired reflected the real conditions and traffic typology. In the following Figure 7 an image acquired from one of the installed sensors shows the monitored road with the RoIs highlighted for each lane. For the upper lane, vehicles are first passing over $R_{1}$, then after a time *t* they will pass over $R_{2}$. The distance between the two RoIs is fixed and known, thus a computation can be performed to establish the length and speed classes of each vehicle following the algorithm presented in Section 3.2.2.

**Figure 8:**
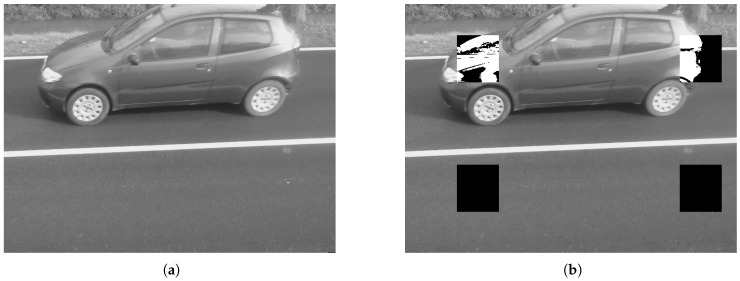
Traffic flow analysis: detected vechicle from sensor test set-up (**a**) and the same frame processed with the RoIs highlighted (**b**).

In Figure 8 the example of a detected vehicle with the corresponding RoIs highlighted is shown.

The frame rate of the camera sensor is known and, considering the width of the RoI, it allows to catch the entrance of a car and the empty space between two passing cars with a safe margin. Thus, each vehicle can be detected individually, and independently from its length. As reported in the algorithm in Figure 3, the main variable is the time *t* occurring between the transition from occupation of $R_{1}$ to occupation of $R_{2}$ and then to the exit of $R_{2}$. The analysis of the two RoIs is performed independently, so that one can result in an *occupied* status while the other may result *free*.

The RoI for the traffic monitoring are two for each lane, and the distance among each RoI in the same lane is measured at the road surface level and used in order to compute the vehicles speeds and length classes. Three speed classes and three length classes were used. Test sequences have been acquired in real traffic conditions and then used for testing the algorithm. The ground-truth total for these sequences was the following: 124 vehicles transited (70 along the lower lane, 54 on the upper lane) and having the following length classes: 11 with length between 0 and 2 m (7 lower lane, 4 upper lane); 98 with length between 2 and 5 m (55 lower lane, 43 upper lane); 15 with length 5 and more metres (8 lower lane, 7 upper lane).

It is important to note, for the following results, that the lengths were inferred on the basis of the recognition, by a human observer by sight, of the specific car models. Moreover, the algorithms compute a speed class estimate, for this data the ground truth is based on preliminary tests made on cars which were equipped with a GPS and recorded their own speeds. The total classification results are shown in Table 3.

As it was expected, the results on the upper lane were slightly less precise; nevertheless the global performance for this test has to be considered positive, yielding a percentage of more than 95% of correct detections. Furthermore, a classification for the length classes is reported, where the 3 nominal classes for length are shown in Table 4. As already stated, the lengths have been extracted from car manufacturer data. Another test was made regarding speed classes, but in this case we did not have a complete ground-truth data because the estimates were feasible only by eye-sight, thus we did decide not to show the outcome of this test.

In order to show the relevance of our proposal, we report, in the following Table 5, a  comparison with other state of the art algorithms, evaluated with respect to correct identification rate (i.e., a performance index), and the computational power used for these solutions. Obviously, an effective and complete comparison is impossible, due to the different tasks, goals, algorithms, case studies used by each cited work.

### 4.2. Evaluation of Middleware Capabilities

This section reports the ICSI Middleware performance evaluation in a laboratory testbed. The main purpose of such a campaign is to evaluate the middleware capabilities in a controlled environment. In the following of the section, the laboratory testbed is first described, then the middleware performance are reported in terms of event notification delay, by considering both event composition and data encoding techniques required for the transmission of M2M messages. Moreover, since ideally the middleware is targeted to constrained devices, a feasibility assessment is reported considering the actual networking component board introduced in Section 3.1.

**Figure 9:**
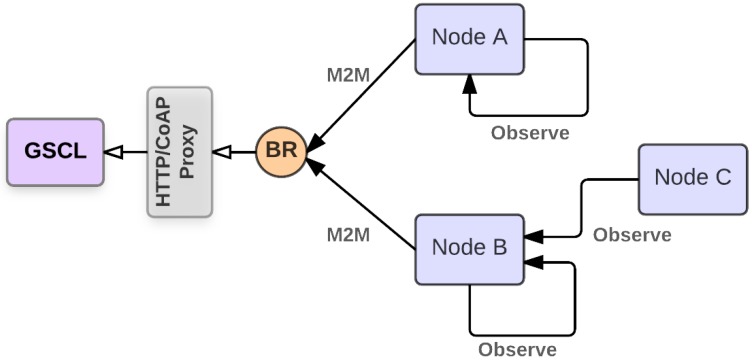
Experimental setup in the laboratory testbed.

The testbed setup is reported in Figure 9. It is mainly composed of three visual sensor nodes, a border router (BR) device able to gather data from the roadside network, a proxy able to translate CoAP messages in the HTTP format, and system able to receive HTTP messages from the proxy while supporting the Gateway Service Capability Layer (GSCL) component defined in the ETSI M2M architecture. In the picture the arrows labeled with the Observe method describe the monitoring relationships involving the three visual sensor nodes. In detail Node A is configured to monitor only local events, while Node B monitors event locally and receives event notifications generated by Node C. In Node B an event composition is performed, the basic events are aggregated by the RPE and sent to the GSCL through the proxy as new aggregated resource. The proxy and the GSCL are hosted on the same laptop. The measured transmission latency in event notification is reported in Table 6, it includes the time required to: (i) compose the event, (ii) compress and send the event as M2M message, (iii) convert the in-network message in the HTTP format before sending it to the GSCL component. In the table the overall event notification delay is reported as a function of the message dimension, considering the number of data packets in which it must be divided. As it is easy to expect, the delay increases as a function of the message dimension, and it is mainly due by transmission and encoding latencies, while with the implemented event composition logic (weighted averaging function) the RPE delay is negligible. Increasing the size of the message bigger delays can be experienced. However, the resulting delay is fully compatible with the dynamics of the considered ITS applications.

The middleware feasibility assessment of the proposed middleware solution has been analyzed for the networking component device, the SEED-EYE board [47]. Such a device is characterized by 512 Kbyte of Flash memory and 128 Kbyte of RAM memory. The whole middleware requires 44.80% of the ROM memory and only 26.50% of the RAM, leaving a significant space available for the user-defined event composition functions.

### 4.3. Experimentation in the Field

The final deployment of the visual sensor network has been arranged in a specific area of the city of Pisa featuring an important commuter parking lot and a main road for accessing both the parking facility and the city center. In order to have an autonomous system, all the mounted sensor nodes were equipped with photovoltaic panels and batteries for long term duration.

**Figure 10:**
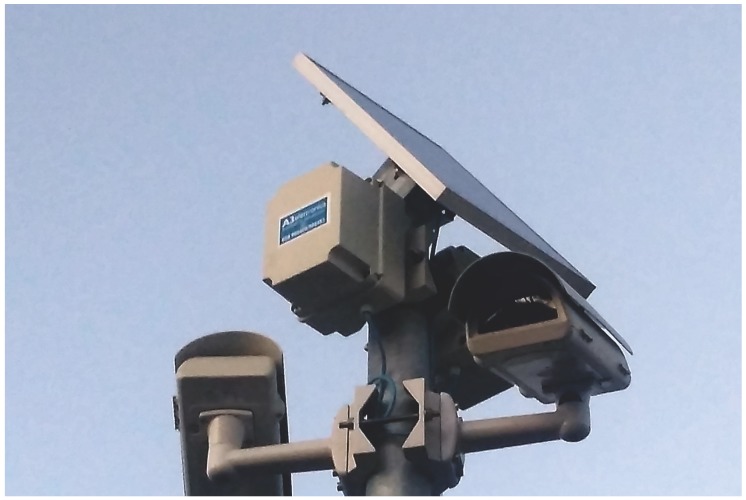
Picture showing the final field test installation.

In Figure 10 two of the sensor nodes of the installed network are shown. It is important to notice that in the installation only normal poles were used for fixing the sensors that, being completely wireless, did not require any further work for cabling. The full installation required less than two days of work of a team constituted by 2 technicians and 2 computer scientists taking care of sensor fixing and configuration.

A long term monitoring has been performed on both traffic flow and parking lot monitoring, the results span over a period of two months. Each of the test sets used different metrics and evaluation methods, in particular, the parking lot tests analysed the occupancy ratio daily rates and trends, while the flow monitoring tests analysed the traffic flow rate on daily and global basis as well as the average speeds and the cumulative vehicle count curves (i.e., N-curves).

#### 4.3.1. Parking Lot Monitoring Tests

A total number of 71 slots (66 regular, 4 disabled people, 1 e-car charging) were monitored by installing 12 sensor nodes. About 20 slots were in the field of view of more than one sensor in order to test and validate cooperative sensing functionalities of ICSI middleware. In particular, for each of these slots, event composition was performed aggregating the measures produced by each sensor in charge of its monitoring. As in Section 4.2, weighted average was used in aggregation: each sensor contributed to the average with a weight proportional to the area in pixel of the region in the image corresponding to the monitored slot. By evaluation of the log of the network, it resulted that event composition leads to a reduced number of fluctuation, allowing to filter out events due to temporary occlusions in the field of views. In addition, event composition allowed to cope with failure of one of the nodes. Indeed, tests have been executed switching off artificially a sensor in the network. The visual sensor network was able both to reconfigure itself building new routes when needed as well as for publishing aggregated notification regarding parking lot status.

In the following Table 7 a resume of the cooperative monitoring performance is reported. The monitoring regards the slots which are surveilled by two sample cameras (i.e., 12 slots), comparing each individual camera results (i.e., columns CAM A and CAM B) only for the slots monitored by both, versus the cooperative weighted results (i.e., shown in the column COOP.). As it can be seen the worse results from single cameras are heavily increased, as the error rates drop from 6% and almost 5% down to only 1% of errors, counting on a total of 78,840 events on which the occupancy detection algorithm is called, computed as the product of every slot in each acquired frame.

**Figure 11:**
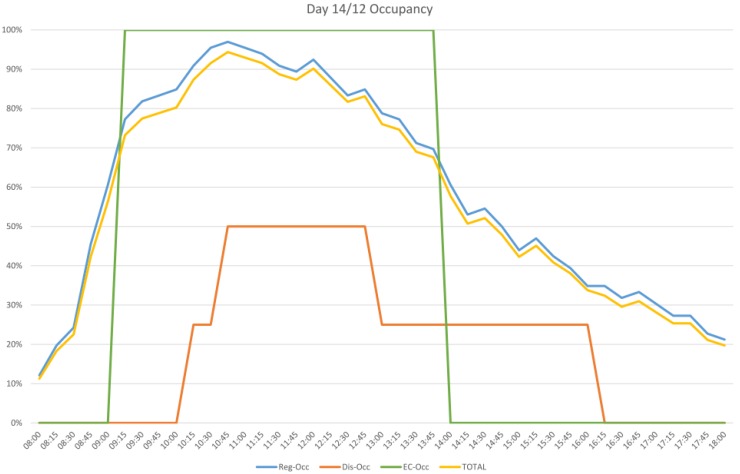
Percentage of parking occupancy on December 14.

Besides these technological consideration, the network was able to collect useful data for understanding the dynamics of parking lot access and usage. A typical working day scenario is reported in Figure 11 where the occupancy status recorded every 15 min is shown for each of the slots categories (i.e., Reg-Occ: regular slots; Dis-Occ: disabled people slots; EC-Occ: e-Charging slots; TOTAL: Total % of available slots).

A more extensive assessment of these results is given by the monthly aggregated data, for the whole months of November and December until the last day of the year. Obtained results confirm the usage of the selected parking lot as a typical long-stay swapping parking used also for mobility exchange reasons (e.g., fast bus stop link to centre and train station). In fact, the occupancy ratio reaches its highest values early and quickly in the morning and only decreases slowly in the afternoon to get to its minimum close to the office closing time. Another relevant information regards weekends and Christmas holiday period (last week of December) low usage of the parking (i.e., never exceeding 35% of the capacity). One more suggestion comes looking at the peculiar behaviour during the week before Christmas (i.e., December 21–23); there is yet a decrease in the total occupancy, and an increase in the latter time slots (e.g., 14–18), a possible explanation of this can be inherent to a higher flow of people staying longer in the city centre for Christmas shopping.

#### 4.3.2. Traffic Flow Monitoring Tests

The long term flow monitoring tests were performed along Via Pietrasantina (the north access way to the city of Pisa). Data acquired covered different aspects: the amount of vehicles, the average speed categories detected, and the aggregation of these data on time slots and daily basis. The first data analysed is the traffic flow rate on a single working day. Data are evaluated on a 15 min basis.

As it was expected the flow ratios, in a normal working day, have the highest values in the early morning hours to quickly decrease at around 10:00, to increase again during lunch-time (i.e., that is also around closing time of schools). A rapid decrease happens around 14:00, increasing again but to a slightly lower value, after 16:00 for the closing office time, which is more distributed in time (e.g., spanning from 16:00 till 20:00). Another interesting data is the total amount of vehicles: in that day the amount was 2749, which considering the over 95% correct identification ratio confirms to be in range of the typical working day data available from the Pisa mobility agency.

**Figure 12:**
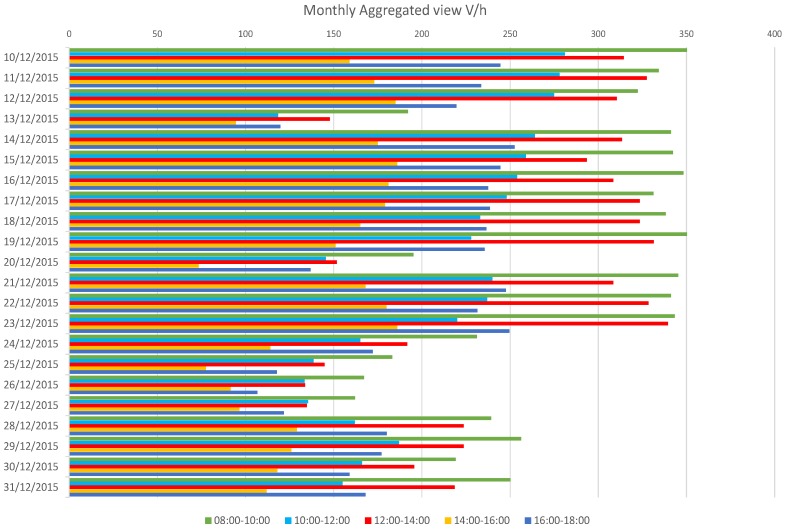
Aggregated vehicles per hour data by time slots for the latter 3 weeks (reduced only for a better readability) of December 2015.

The comprehensive results obtained during the two months monitoring are given, by means of monthly views of the vehicles-per-hour data aggregated by time slots (example in Figure 12 showing the month of December).

A brief analysis of the results brings to some remarks: differently from the parking lot tests, the traffic flow shows large decrease only on Sundays and holidays, while Saturdays see a traffic flow comparable to other working days. Sundays and holidays (i.e., 8 December, 25 and 26 December) report the average traffic flow ratio to drop more than 50%. Moreover, alike the parking lot scenario, during Christmas holidays (i.e., after 23 December) there is a decrease around 30% of the traffic flow.

## 5. Conclusions

In this paper, a prototype of visual sensor network has been presented where each camera-equipped node embeds special vision logics to understand urban mobility and extract relevant real-time data for its analysis. Globally, the network is endowed with an IoT middleware that enables cooperative sensing by offering the possibility to perform event composition at network level. In this way, the network is insensitive to hardware failure as well as to temporary occlusion in the field of view of some nodes. Besides the lab, the capability of the network has been demonstrated in a field test that has highlighted the suitability of the proposed solution in dealing with parking and traffic flow monitoring and providing high quality real-time information. Indeed, the network was able to capture and measure variations in urban mobility produced under special circumstances such as festivities; such altered patterns would have been difficult to collect at large scale without an IoT solution for the smart city as the one proposed in this paper. Furthermore, thanks to great applicability of vision, the visual sensor network can support the collection of data in other smart city contexts simply by extending the already developed vision logics.

## Figures and Tables

**Table 1:** Performance of parking lot monitoring of 5 separate devices.

	Monitored Slots	Total Frames	False Hit Events	Missed Events	Total FP	Total FN	Error Rate
**CAM A**	23	5357	10	24	238	594	0.675%
**CAM B**	22	5145	8	22	285	693	0.864%
**CAM C**	17	5260	7	14	156	396	0.617%
**CAM D**	16	5225	6	8	222	269	0.587%
**CAM E**	15	5305	6	5	211	197	0.513%

**Table 2:** Comparison of related work.

Reference	Error Rate (%)	Features
Wu et al., 2007 [61]	6.5	*Color*
Sastre et al., 2007 [22]	2.2	*Gabor filters*
Bong et al., 2008 [62]	7.0	*Color*
Hichihashi et al., 2009 [63]	2.0	*PCA*
Huang and Wang 2010 [17]	1.2	*Color*
DeAlmeida et al., 2015 [25]	0.4	*Texture*
Amato et al., 2016 [27]	0.4	*CNN*
Proposed method	0.65	*Edge and color*

**Table 3:** Classification performance of the traffic flow monitoring.

	Total	Lower Lane	Upper Lane
**Total transited vehicles**	124	70	54
**Correctly identified vehicles**	118(95.2%)	69 (98.6%)	49 (90.7%)
**False positives**	3 (2.4%)	1 (1.4%)	2 (4%)

**Table 4:** Example of classification with respect to length (*ℓ*). Analysis performed for lower lane data.

Length Class	Ground Truth	Correct Class.	False Positive	Efficiency
ℓ≤2 m	8	8	0	100%
2<ℓ≤5 m	57	56	1	96.6%
ℓ>5 m	5	5	0	100%
**TOTAL**	70	69	1	97.2%

**Table 5:** Comparison of performances and computational power for different algorithms.

	Performance	Hardware/Processing Notes
**Messelodi et al., 2005 [35]**	82.8%	*Hardware not reported*
		**not an embed. platform**
**Ottlik&Nagel 2008 [36]**	83%	*Hardware not reported*
		**off-line processing**
**Saunier&Sayed 2006 [37]**	88.4%	*Hardware not reported*
		**off-line processing**
**Chen et al., 2011 [38]**	97%	DM642 **DSP-based**
		**embed. platform**
		600 **MHz-**32 **MB DRAM**
**Rad&Jamzad 2005 [39]**	96%	**Pentium II** 800 **MHz**
		**processing 11fps**
**Lai et al., 2008 [40]**	89%	*Hardware not reported*
		**but processing** 2.5 **fps**
**Semertzidis et al., 2010 [41]**	91%	*Hardware not reported*
		**but processing <**5 **fps**
**Chen et al., 2012 [42]**	96%	*Hardware not reported*
		**off-line processing**
**Proposed method**	95.2%	**ARM Architecture**
		454 **MHz-**64 **MB SDRAM**

**Table 6:** Event notification latency.

Message Size [Bytes]	Number of Messages	Sensor to GSCL [ms]
104	2	176.01 ± 0.27
155	3	227.79 ± 0.42
228	5	287.06 ± 0.38

**Table 7:** Cooperative monitoring results.

	CAM A	CAM B	COOP.
ERRORS	4870	3674	804
%	**6.2%**	**4.7%**	**1.0%**
